# Abnormal Trabecular Bone Score, Lower Bone Mineral Density and Lean Mass in Young Women With Premature Ovarian Insufficiency Are Prevented by Oestrogen Replacement

**DOI:** 10.3389/fendo.2022.860853

**Published:** 2022-05-19

**Authors:** Navira Samad, Hanh H. Nguyen, Hikaru Hashimura, Julie Pasco, Mark Kotowicz, Boyd J. Strauss, Peter R. Ebeling, Frances Milat, Amanda J. Vincent

**Affiliations:** ^1^ Department of Endocrinology, Monash Health, Melbourne, VIC, Australia; ^2^ Department of Medicine, School of Clinical Sciences, Monash University, Melbourne, VIC, Australia; ^3^ IMPACT - Institute for Physical and Mental Health and Clinical Translation, Deakin University, Geelong, VIC, Australia; ^4^ Department of Medicine, Western Health, The University of Melbourne, Melbourne, VIC, Australia; ^5^ Department of Epidemiology and Preventive Medicine, Monash University, Melbourne, VIC, Australia; ^6^ University Hospital Geelong, Barwon Health, Geelong, VIC, Australia; ^7^ Department of Endocrinology and Diabetes, University Hospital Geelong, Barwon Health, Geelong, VIC, Australia; ^8^ Division of Diabetes, Endocrinology and Gastroenterology, School of Medical Sciences, Faculty of Biology, Medicine and Health, The University of Manchester, Manchester, United Kingdom; ^9^ Hudson Institute of Medical Research, Melbourne, VIC, Australia; ^10^ Monash Centre for Health Research and Implementation, Monash University, Melbourne, VIC, Australia

**Keywords:** premature ovarian insufficiency, osteoporosis, bone mineral density, trabecular bone score, body composition, appendicular lean mass

## Abstract

**Background:**

Low bone density (BMD) and fractures commonly affect women with premature ovarian insufficiency (POI). However, bone microarchitecture and body composition data are lacking.

**Objective:**

To assess and characterise musculoskeletal phenotype and effects of oestrogen replacement therapy (ERT) in women with POI.

**Method:**

Cross-sectional and longitudinal studies of 60 normal karyotype women with POI, aged 20-40 years, from 2005-2018. Dual x-ray absorptiometry (DXA)-derived spinal (LS) and femoral neck (FN) BMD, trabecular bone score (TBS), appendicular lean mass (ALM), total fat mass (TFM), and fracture prevalence were compared with 60 age-, and BMI-matched population-based controls. Longitudinal changes in bone and body composition variables and ERT effects were analysed using linear mixed models over a median duration of 6 years.

**Results:**

Women with POI were subdivided into spontaneous (s)-POI (n=25) and iatrogenic (i)-POI (n=35). Median(range) age of POI diagnosis was 34 (10-40) years with baseline DXA performed at median 1(0-13) year post-diagnosis. ERT was used by 82% women (similar for both POI groups). FN-BMD were lowest in s-POI (p<0.002). Low TBS was more common in s-POI [(44%), p=0.03], versus other groups. LS-BMD and ALM were lower in both s-POI and i-POI groups than controls (p<0.05). Fracture prevalence was not significantly different: 20% (s-POI), 17% (i-POI), and 8% (controls) (p=0.26). Longitudinal analysis of 23 POI women showed regular ERT was associated with ALM increment of 127.05 g/year (p<0.001) and protected against bone loss. However, ERT interruption was associated with annual reductions in FN BMD and TBS of 0.020g/cm^2^ and 0.0070 (p<0.05), respectively.

**Conclusion:**

Deficits in BMD, trabecular microarchitecture, and lean mass were present in women with POI. However, regular ERT protected against declines in bone variables, with an increase in ALM. Assessment of skeletal and muscle health, and advocating ERT adherence, is essential in POI to optimise musculoskeletal outcomes.

## Introduction

Up to 4% of women (1) are affected worldwide by Premature Ovarian Insufficiency (POI), a condition in which cessation of female gonadal function occurs before age forty years. POI is broadly categorised into spontaneous and iatrogenic POI. Spontaneous POI (s-POI) is idiopathic in most cases, but can be associated with chromosomal and genetic defects, environmental factors, autoimmune diseases (most commonly thyroid and adrenal disease) and various infections. In contrast, iatrogenic POI (i-POI) is secondary to medical and surgical causes such as chemotherapy, ionising radiotherapy, or bilateral oophorectomy (2).

POI is associated with increased mortality and morbidity due to the loss of oestrogen’s beneficial effects on various tissues and organs in the body, especially cardiovascular, neurocognitive, and musculoskeletal systems (3). Prompt diagnosis and management of POI and its sequelae can significantly reduce morbidity and mortality in this group.

While there is no unified definition of osteoporosis and osteopenia in adults aged < 50 years, researchers have indicated low bone mass and osteoporosis prevalence to be around 8-15% in women with POI (4, 5); the risk is dependent on the degree and duration of oestrogen deficiency. There is preferential loss of trabecular bone, leading to an almost 26% reduction in lumbar spine (LS) bone mineral density (BMD) compared with age-matched population controls (6). Although the exact prevalence of fractures remains unknown, it is anticipated to be high, with fractures occurring at a younger age (7). Small studies show that oestrogen can maintain and restore bone mineral density (BMD) in POI (8, 9) but the optimal formulation, dose and type of oestrogen replacement therapy (ERT) is unknown. Defining the musculoskeletal phenotype in POI, including BMD, fracture prevalence, body composition and effects of oestrogen on these indices, is important for the optimal management of these women.

Dual-energy X-ray absorptiometry (DXA) is routinely used to assess fracture risk, but it has disadvantages, including two-dimensional projection of areal-BMD (a-BMD), inability to differentiate between trabecular and cortical bone, and underestimation of BMD in women with short stature, such as Turner syndrome (TS). More recently, techniques such as trabecular bone score (TBS), can provide additional information relating to trabecular microarchitecture and are independent predictors of fracture risk.

TBS is computed from LS DXA images and constitutes a textural representation that assesses pixel grey‐level variations in the LS, providing an indirect analysis of trabecular microarchitecture (10). Despite data from longitudinal studies in older populations showing TBS predicts fractures independently of BMD and FRAX^®^ (11–14), data are limited in women with POI, in premenopausal women, and in younger men. In a small cohort of women with TS, a recognised genetic cause of POI, our group has previously reported an age-related decrease in TBS (15).

With recent insights gained from muscle-bone cross signaling (16), it is now evident that muscle plays an essential role in maintaining and preserving skeletal health. Studies in typical menopausal women suggest an accelerated decline in muscle mass, increase in abdominal adiposity, and reductions in lean body mass (LBM) due to oestrogen deficiency, with preservation in women using ERT (16). However, data in women with POI are scant and there is a growing need to analyse changes in body composition, as well as in bone variables in women with POI.

The aims of this study are to:

1) compare clinical and densitometric data, including skeletal and body composition assessments, between women with normal karyotype POI and age-matched healthy Australian women (controls) from the population-based Geelong Osteoporosis study (GOS), 2) analyse the longitudinal changes in the skeletal and body composition parameters in women with POI; and 3) investigate fracture prevalence in women with POI.

## Methods

### Study Participants

#### Women With POI

**Figure 1:**
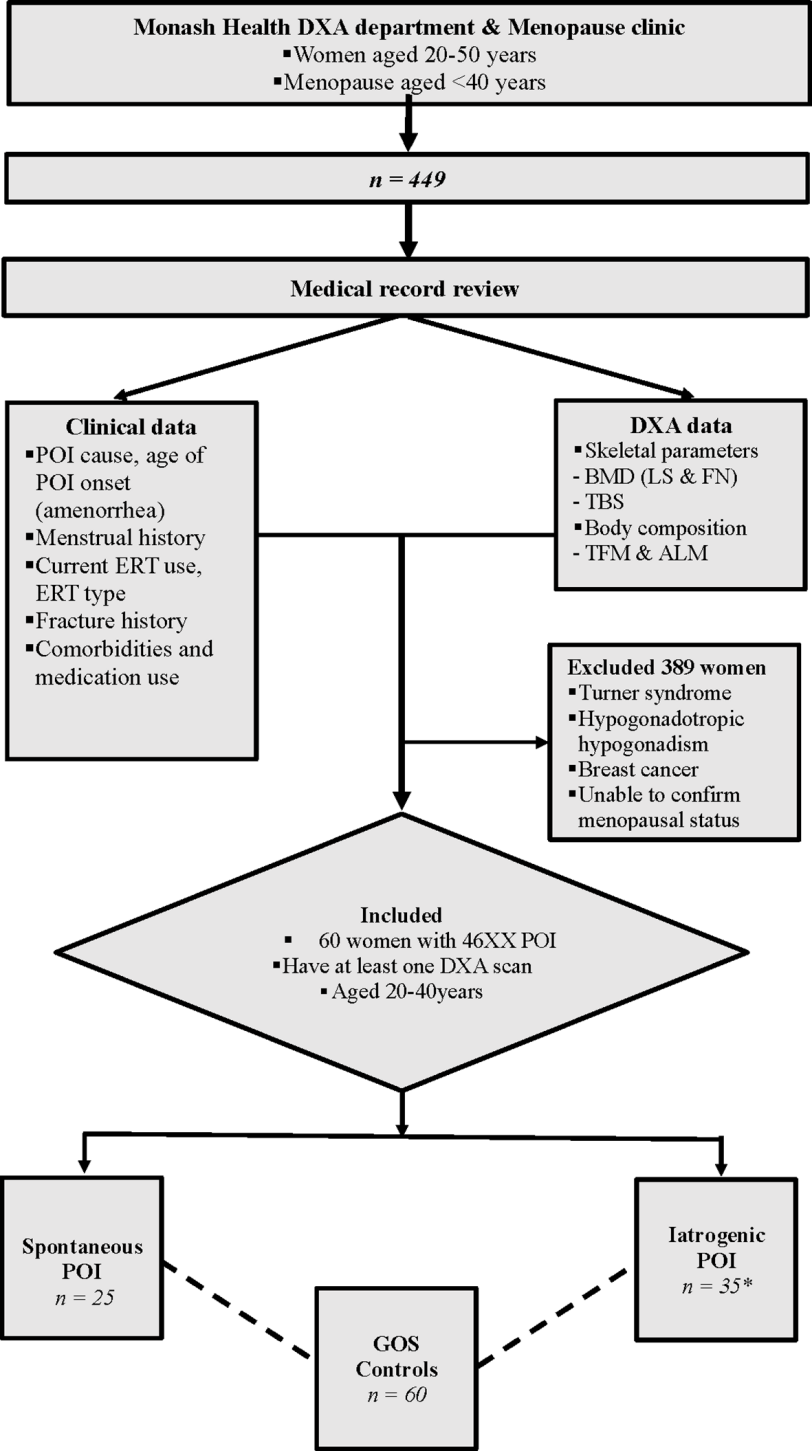
Study scheme. The bone & body composition parameters were compared between 25 s-POI and 35 i-POI patients from the Monash Health POI cohort and 60 age & BMI matched controls from the GOS. *27 patients had non-breast cancer malignancy, 27 patients had oophorectomies and 17 had chemo ± radiotherapy. DXA, Dual energy Xray Absorptiometry; POI, Premature ovarian insufficiency; ERT, Estrogen replacement therapy; a-BMD, aerial bone mineral density; LS, Lumbar spine; FN, Femoral neck; TBS, Trabecular bone score; TFM, Total fat mass; ALM, Appendicular lean mass; GOS, Geelong Osteoporosis Study.

This retrospective, observational study included women aged 20 to 40 years who had POI and underwent one or more DXA scans between January 2005 to May 2018 at Monash Health, Clayton, Victoria, Australia. Participants were identified from the Monash Health Early Menopause Clinic, in addition to a comprehensive search of the bone density unit’s database for all women documented as having hypogonadism, and aged 20 to 40 years. Women with TS, hypogonadotropic hypogonadism, or a history of breast cancer were excluded (**Figure 1**). The study was approved by the Monash Health Human Research Ethics Committee (Reference number 07062A).

During the study period, all DXA scans for the POI women were performed at Monash Health on a single Lunar Prodigy device (GE Healthcare, 133 Piscataway, NJ) using software version 11 to measure skeletal and body composition parameters. Baseline and serial DXA scans conducted during the study period for each woman were included in the analyses.

For skeletal assessments, all women with POI had BMD (g/cm^2^) measured at the lumbar spine LS (L1-L4) and femoral neck (FN). TBS measurements were obtained from DXA spinal images using TBS iNsight Software (version 3.0.2.0; Medimaps, Geneva, Switzerland). The coefficients of variation CV for TBS and BMD using a LS phantom measured daily on the GE Lunar Prodigy were 0.94% and 0.92%, respectively.

On the day of the DXA scan, each participant’s height (m) and weight (kg) were measured with a wall-mounted stadiometer and electronic scale, respectively. Body composition measures including total fat mass (TFM) (g) and appendicular lean mass (ALM) (g) were were derived from the whole-body scan and both parameters were also adjusted for height to yield FMI (TFM/ht^2^ in g/m^2^) and ALMI (ALM/ht^2^ in g/m^2^)), respectively. The CV for percentage body fat of a total body phantom measured weekly was 3.11%.

Data regarding age and cause of POI, comorbidities, and medication use (including ERT were verified from hospital medical records and the adult menopause clinic database. Moreover, details on ERT use, including age at initiation and ongoing use at the time of DXA scanning, were also retrieved. A history of all documented fractures was established from medical records or by radiologically proven fractures from the hospital’s radiology database.

#### Controls

The data for control participants were obtained from the Geelong Osteoporosis Study (GOS), an Australian longitudinal, population-based study, which aims to define the epidemiology of osteoporosis and fracture risk in men and women; a comprehensive description of the study has been provided elsewhere (17). A Lunar DPX-L (Lunar; Madison, WI, USA) was used for the women’s skeletal and body composition assessments, including LS BMD, FN BMD, TFM and ALM.

TBS (L1-L4) was retrospectively calculated from the same DXA spinal images using TBS iNsight software (version 2.2; Medimaps Group, Geneva, Switzerland). The coefficient of variation (%) for TBS was 1.93%. Body weight (± 0.1 kg) was measured using electronic scales and height (± 0.1 cm) using a wall-mounted stadiometer. Fracture history was determined by self‐report and confirmed radiologically where possible. The provision of de-identified data for this study was approved by the Human Research Ethics Committee of Barwon Health.

### Statistical Analysis

The software program IBM SPSS Statistics v23 (IBM Corp, Armonk, NY) was used to conduct all cross-sectional statistical analyses, except when Stata v14.2 statistical software (StataCorp, College Station, TX) was used for mixed model analysis to assess longitudinal densitometric changes. A *p* value <0.05 was considered statistically significant.

#### Cross-Sectional Data

For cross-sectional analysis, categorical data for women from both groups were expressed as percentages and continuous data as mean ± SD or median and 25^th^ & 75th percentiles, depending on the data distribution. The distribution of data was explored using the Shapiro-Wilk test. TBS was analysed as both continuous and dichotomous variables using established thresholds for abnormal value, TBS <1.302 (18) where partially degraded and degraded TBS was grouped as low TBS. Differences between groups were determined using the ANOVA for normally distributed continuous variables with Bonferroni’s method for *post-hoc* analyses; the Kruskal-Wallis test was used for nonparametric variables with the Mann-Whitney U for pairwise comparison. The Chi-square and Fisher’s exact test were used for categorical variables.

#### Longitudinal Data

Women with POI who had serial DXA scans were included in the longitudinal analysis. Linear mixed model analysis was used to determine the mean change per year (95%CI) in bone (TBS, LS, and FN BMD) and body composition parameters (TFM, FMI, ALM and ALMI) over time, with time defined as the number of years since baseline DXA. To determine the effect of ERT use on longitudinal changes, POI women with continued ERT use (defined as ERT use documented at the time of DXA scans) and interrupted ERT use were analysed separately, and data presented in two-way scatterplots with overlaid linear prediction plots.

## Results

### Baseline Characteristics

Sixty women with POI and 60 age- and BMI-matched control participants were identified. For cross-sectional analysis, women with POI were subdivided into two groups i) spontaneous POI (s-POI, n=25); ii) iatrogenic POI (i-POI, n = 35). In the i-POI group 27 patients had non-breast cancer malignancy, 27 patients had bilateral oophorectomies and 17 had chemo ± radiotherapy. The baseline characteristics of participants are shown in **Table 1**.

**Table 1:** Demographics and baseline skeletal & body composition analysis of POI women and controls.

	Control n=60	s-POI n=25	i-POI n=35	P Value
**Age (years)**	34 (30-38)	35 (29-38)	33 (30-38)	0.72^a^
**Ethnicity**
Caucasian	59 (98%)	17 (68%)	29 (82%)	**<0.001^b^**
Asian	0	5 (20%)	6 (17%)	NA
Black	0	0	1 (3%)	NA
Hispanic	0	1 (4%)	0	NA
**BMI (kg/m^2^)**	25.84 (22.48, 30.83)	24.80 (21.50, 27.30)	25.70 (22.50, 31.30)	0.25^a^
**Height (m)**	1.66 (1.61,1.70)	1.58 (1.55, 1.68)	1.61 (1.58, 1.66)	**0.002^a^**
**Fracture history**	5 (8%)	5 (20%)	6 (17%)	0.275. ^b^
ERT use^*^	NA	15 (68%)	29 (91%)	0.07^b^
**Bone Density**
LS BMD (g/cm^2^)	1.26 (01.18, 1.39)	1.07 (0.96, 1.20)	1.13 (1.05, 1.28	**<0.001^a^**
FN BMD (g/cm^2^)	1.04 ± 0.13	0.91 ± 0.14	1.00 ± 0.16	**0.002^c^**
**TBS**
TBS score	1.40 ± 0.11	1.36 ± 0.12	1.37± 0.10	0.19^a^
Low TBS (n)%	12 (20%)	11 (44%)	6 (17%)	**0.03^b^**
**Body Composition**
ALM (g)	19627.50 (17718.00, 21360.00)	15891.00 (14063.50, 17247.00)	16952.00 (14613, 19029.50)	**<0.001^a^**
ALMI (g/m^2^)	7077.83 (6452.79, 7740.68)	6172.69 (5472.46, 6638.57)	6154.71 (5645.69, 7100.59)	**<0.001^a^**
TFM (g)	26856.50 (17984, 40504)	20820.00 (18617.27, 31562.50)	26178.00 (20628, 35213)	0.29^a^
TFMI (g/m^2^)	9823.08 (6404.68,14134.91)	8944.46 (7598.21, 1270.57)	9537.52 (7020.77, 13806.36)	0.28^a^

*Total n=54; s-POI=22, i-POI n=32; data not available in n=6.

#LS BMD after excluding women with fracture (n=2); controls 1.40 (1.33,1.46); s-POI 1.32 (1.27, 1.46); i-POI 1.37 (1.32, 1.43); p<-.001.

!FN BMD after excluding women with fracture (n=2); controls 1.04 (± 0.13); s-POI 1.32 (± 0.14); i-POI 1.00 (± 0.16)); p=-.005.

BMI, body mass index; ERT, estrogen replacement therapy; BMD, bone mineral density; LS, lumbar spine; FN, femoral neck; TBS, trabecular bone score; ALM, appendicular lean mass; ALMI, appendicular lean mass index; TFM, total fat mass; TFMI, total fat mass index.

p values reported using: ^a^Kruskal-Wallis test; ^b^ Fisher’s-exact test; ^c^ANOVA; boldface indicates p values that are statistically significant. Data are presented as mean ± SD when normally distributed or median and interquartile range (IQR 25,75) when not normally distributed, except for age presented as median (range).

Across the three groups, BMI and age were well-matched. Compared with the control group, participants in the two POI subgroups were shorter and more ethnically diverse (all p<0.05). The median age of POI onset in s-POI and i-POI group was the same, 32 years. Most women with POI were using ERT (81%); menopausal ERT was used by 67% and 78% of women in s-POI and i-POI groups, respectively, and the combined oral contraceptive pill (COCP) was used by 33% and 12% of women in s-POI and i-POI groups, respectively.

In the i-POI group, four patients were smokers, and four had an additional cause for low bone density, including coeliac disease, hyperthyroidism, another autoimmune disease, and chronic renal failure. Five patients had vitamin D deficiency, one had another autoimmune disease in the s-POI group, and none were smokers. Among the POI women, only one patient in the s-POI group who had an L1 vertebral fracture received antiresorptive therapy with denosumab, which commenced six years after the onset of POI and the baseline DXA scan. The age at menarche was available for 12/25 patients in the s-POI, and the median age was 14 years. In the i-POI group, this data was available for 13/35 patients, and the median age was 13 years.

The BMD at LS and FN differed across the three groups (p<0.05) (**Table 1**). Control women had significantly higher LS BMD (g/cm^2)^ [1.26 (01.18, 1.39)] than s-POI [1.07 (0.96, 1.20)] and i-POI [1.13 (1.05, 1.28); although the LS BMD did not significantly differ between the s-POI and i-POI groups (p=0.074). The FN BMD (g/cm^2^) in the s-POI group (0.91 ± 0.14) was also significantly lower than both control (1.04 ± 0.13) and i-POI (1.00 ± 0.16) groups, which both had similar FN BMD (p=0.90). The LS and FN BMD results remain unaltered after excluding two women with vertebral fractures, one each in the control and s-POI groups from the cross-sectional analysis (**Table 1**).

In the POI cohort, the prevalence of DXA defined osteoporosis was 5% (3/60) as defined by the IOF for young adults i.e., T-score < - 2.5 at spine or hip in association with a chronic disease known to affect bone metabolism (19) and 13% had low bone mass according to the ISCD criteria as spine or hip BMD Z-score <-2 (20). The prevalence of fracture in this cohort is described below.

**Figure 2:**
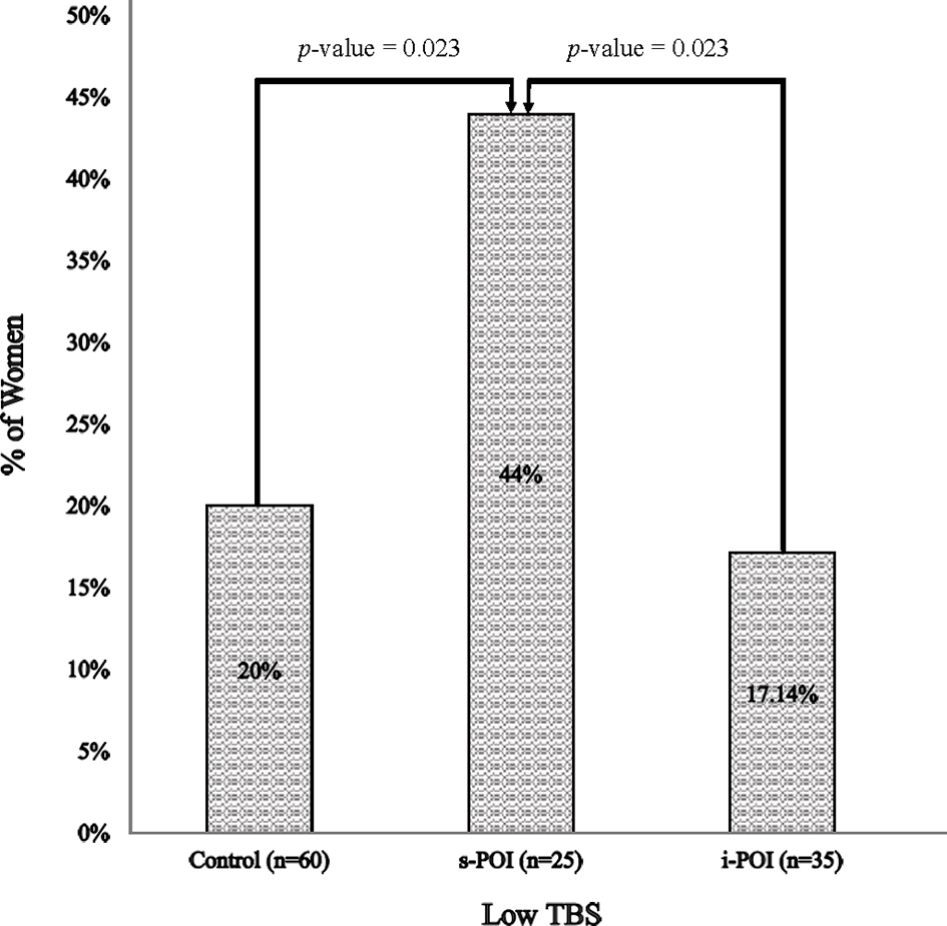
Prevalence of low TBS. Low TBS was defined as TBS <1.302 [including both partially degraded & degraded TBS (18)].

Overall, the prevalence of low TBS differed across the three groups (p= 0.03). The pairwise comparison suggested that the low TBS prevalence was significantly higher in the s-POI group (44%) than the i-POI and control groups (both p < 0.05) (**Figure 2**).

Body composition analysis revealed significantly lower ALM (g) in s-POI [15891.00 (14063.50, 17247.00)] and the i-POI [16952.00 (14613.00, 19029.50)] subgroups than the control group (both p < 0.001); however, ALM did not differ between the two POI groups (p=0.160). No significant differences in TFM were observed across the three groups.

### Longitudinal Study

Longitudinal densitometric data were available for 24 out of 60 POI women; however, one participant receiving antiresorptive therapy with denosumab in the interrupted ERT group was excluded from the final analysis. The median follow-up duration was six years (range, 1 to 12 years), and 75% of women had follow-up data available for at least five years.

Over the follow-up period, 17 women with POI continued ERT; the majority (14/17) used HRT and 3/17 were on combined oral contraceptive pills (COCP). In the interrupted ERT group (*n*=6), 3 women who were not receiving therapy at baseline commenced ERT during follow-up; two women never received ERT; one woman ceased ERT during the study, and the ERT status was unknown for one woman. Longitudinal data were not available for the remaining 36 women because they were either lost to follow-up or did not have a second routine DXA scan at the time of study analysis.

During follow-up, significant longitudinal changes per year were observed in skeletal and body composition variables. Overall, women with POI lost 0.0049 g/cm2 per year in FN-BMD (p = 0.038); additionally, a significant increase in ALM and ALMI was noted overtime at [(71.61 (95% CI: 8.89, 134.32), p=0.025), (33.62 (95% CI:12.50, 54.74), p=0.002)], respectively (**Table 2**). No significant changes were seen in TBS, spine BMD, or fat mass (TFM and FMI).

**Table 2:** Longitudinal changes in bone & body composition outcomes.

Parameter	Δ/Year (95%CI)	p-value
**All POI cases *(n=23)***		
LS BMD, g/cm^2^	0.0012 (0.0025, 0.0049)	0.51
FN BMD, g/cm2	-0.0049 (-0.0096, 0.00027)	**0.038**
TBS	0.00085 (-0.0032, 0.0049)	0.677
ALM (g)	71.61 (8.89, 134.32)	**0.025**
ALMI (g/m^2^)	33.62 (12.50, 54.74)	**0.002**
TFM (g)	51.80 (-324.95, 428.55)	0.79
TFMI (g/m^2^)	23.19 (-112.27, 158.64)	0.74
**POI cases with continued ERT *(n=17)***		
LS BMD, g/cm^2^	0.0019 (-0.0019, 0.0058)	0.32
FN BMD, g/cm^2^	-0.0016 (-0.0038,0.00059)	0.15
TBS	0.0032 (-0.0019, 0.0083)	0.23
ALM (g)	127.05 (68.81, 185.30)	**<0.001**
ALMI (g/m^2^)	47.29 (25.35, 69.23)	**<0.001**
TFM (g)	96.60 (-250.01, 443.21)	0.59
TFMI (g/m^2^)	37.05 (-96.59, 170.69)	0.59
**POI cases with interrupted ERT *(n=5)***		
LS BMD, g/cm^2^	-0.0035 (-.0014, 0.0067)	0.50
FN BMD, g/cm^2^	-0.020 (-0.037, -0.0024)	**0.025**
TBS	-0.0070 (-0.011, -0.0020)	**0.007**
ALM (g)	7.60 (-139.041, 154.25)	0.92
ALMI (g/m^2^)	5.31 (-52.069, 62.68)	0.86
TFM (g)	-590.48 (-1754.61, 573.64)	0.32
TFMI (g/m^2^)	-154.22 (-537.42, 228.99)	0.43

BMI, body mass index; ERT, estrogen replacement therapy; BMD, bone mineral density; LS, lumbar spine; FN, femoral neck; TBS, trabecular bone score; ALM, appendicular lean mass; ALMI, appendicular lean mass index; TFM, total fat mass; TFMI, total fat mass index.

Boldface indicates p values that are statistically significant.

**Figure 3:**
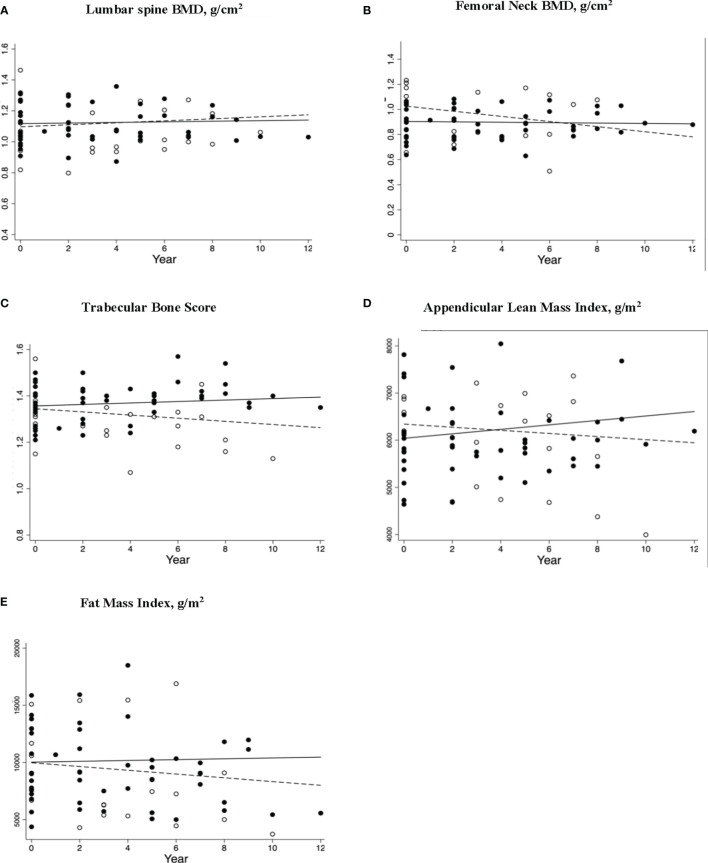
Longitudinal changes in bone and body composition parameters in women (including participant with L1 fracture receiving antiresorptive therapy; n=24) with premature ovarian insufficiency stratified by estrogen replacement therapy (ERT) use. Scatterplots **(A–E)** shows Δ/Year (95%CI) with p values for skeletal and body composition changes for women who had continuous ERT (solid circles) and interrupted ERT (hollow circles) with solid and dashed regression lines indicate the mean annual change in parameter for continued ERT and interrupted ERT, respectively. The respective changes were **(A)** LS BMD Δ/Year (95%CI) 0.0019 (-0.0019, 0.0058) p = 0.32, and -0.0035 (-.0014, 0.0067), p = 0.50 **(B)** FN BMD Δ/Year (95%CI) 0.0016 (-0.0038,0.00059), p = 0.15 and -0.020 (-0.037, -0.0024), p = 0.025 **(C)** TBS Δ/Year (95%CI) 0.0032 (-0.0019, 0.0083), p = 0.23, and -0.0070 (-0.011, -0.0020), p=0.007 **(D)** ALMI Δ/Year (95%CI) 47.29 (25.35, 69.23) p=<0.001 and -590.48 (-1754.61, 573.64), p=0.32 **(E)** TFMI Δ/Year (95%CI) 37.05 (-96.59, 170.69) p=0.59 and -154.22 (-537.42, 228.99) p=0.43 ERT, estrogen replacement therapy; LS, lumbar spine; BMD, bone mineral density; FN, femoral neck; TBS, trabecular bone score; ALMI, appendicular lean mass index; TFMI, total fat mass index.

The POI women who continued ERT had an increment of 127.05 g (95% CI: 68.81,185.30, p<0.001) in ALM and no bone loss from the FN. In contrast, a significant decline in FN-BMD (-0.020g/cm2 (95% CI: -0.037, 0.0030), p=0.025) and TBS (-0.0070 (95% CI: −0.011, −0.0020), p=0.007) was observed in the ‘interrupted ERT’ group. The findings for the Continued ERT and Interrupted ERT groups remained similar, including the woman from the interrupted ERT group receiving antiresorptive therapy with the vertebral fracture in the longitudinal analysis. The scatterplots for bone and body composition parameters with linear prediction plots are shown in **Figure 3**.

### Fracture Analysis

Fracture history was available for all women in POI and the control group. Overall, 5 control (8%), 5 (20%) s-POI and 6 (18%) i-POI women reported fractures with no significant differences observed across the three groups (p=0.275) or between all POI and control women (p=0.15. The mean age in years (range) for sustaining the first fracture was 28.0 (21-37) in the control group, 24.0 (9-34) in the s-POI and 29.5 (16-50) in the i-POI group. For the POI cohort, the time of sustaining fracture since POI onset was unavailable for all the women. In the i-POI group, most women sustained fractures, 67% (4/6) after the POI onset; however, 60% (3/5) women in the s-POI group had fractures before the POI onset.

The fractures in the three groups were mostly non-vertebral with a reported frequency of 4/5 (80%), 4/5 (80%) and 6/6 (100%) in the controls, s-POI group, and i-POI group, respectively. One patient in the controls (vertebral level not specified) and one in the s-POI group had a vertebral fracture (at L1 with 20% reduction in vertebral height). None of the patients in the i-POI group sustained vertebral fractures.

No differences were observed in the skeletal or body composition parameters in the fracture and non-fracture groups in the POI cohort. Moreover, fracture rates were similar between women using ERT at the time of assessment compared with those who were not using ERT.

## Discussion

This study demonstrated that women with s-POI had the worst skeletal outcomes, including the lowest BMD at both the hip and spine and highest prevalence of abnormal TBS compared with women with i-POI or control women. Moreover, women in both POI subgroups demonstrated significantly lower lean mass (ALM and ALMI) regardless of cause. A 0.62%/year decline in FN-BMD was observed over 12 years, whereas no significant change was observed in LS-BMD, TBS, or FMI. Continued ERT use conferred a protective effect on both bone and body composition indices, with stable FN BMD and an increase in appendicular lean mass. Furthermore, a significant reduction in TBS of -0.51% was observed in POI women with interrupted ERT use.

An association of hypoestrogenism with impaired bone health has long been established, where researchers have focused on the impact on BMD. Congruent with previous studies of women with various causes of POI (5, 21, 22), our results also demonstrate lower LS and FN BMD in women with POI compared with control women. The reported prevalence of osteoporosis and low bone mass is consistent with the 8-15% reported in previous studies (4, 5).

Our novel finding of increased prevalence of abnormal TBS in s-POI women with normal karyotype is similar to data previously described by our group in a study of 60 women with Turner syndrome (TS), a genetic cause of POI, which showed that a third of these women had abnormal TBS (15). TBS provides an additional tool to predict fracture risk in older adults, including postmenopausal women. However, studies investigating TBS characteristics and its utility in predicting fracture risk in younger populations are limited. The natural trajectory of TBS can be delineated by a few small studies in pediatric and adolescent populations; Shawwa et al. concluded that TBS starts to increase from Tanner stage III in girls (23) and Dowthwaite et al. reported that the mean comparable adult TBS is attained by 12 months post-menarche (24). In a recent study of 565 participants, including 296 females aged between 4-19 years, Guagnelli et al. found that the TBS was highest for 18-year-old females when corrected for bone age, with an average score of 1.701 (25). Most studies in healthy premenopausal women aged between 20-50 years have described a slow decline (26–30) in TBS except for a North American study reporting stable TBS values in women aged 30-45 years, followed by a decrease thereafter (31). Studies assessing TBS in young females with anorexia nervosa have found a positive correlation between TBS and menstrual status (32, 33). The high prevalence of abnormal TBS in POI cohorts suggests an accelerated loss of microarchitectural integrity due to prolonged hypoestrogenism. It is known that the diagnosis of s-POI is often delayed due to its more insidious presentation, leading to late oestrogen commencement, which may explain the higher rates of abnormal TBS compared with those with i-POI in our study (34, 35).

Skeletal parameters remained stable in those women with POI who continued ERT, consistent with previous studies of both postmenopausal (36) and POI women (8, 9). Adverse bone outcomes were observed in POI women who did not use ERT regularly including a reduction in TBS. In contrast, a study of 60 women with TS aged between 20-50 years (15), showed that TBS remained stable over 10 years with ongoing use of ERT. Our findings affirm the role of oestrogen in maintaining BMD and microarchitecture.

Studies of body composition changes in postmenopausal women show increases in body fat mass with the accumulation of central and visceral adiposity, and a decline in appendicular lean body mass (37–40). A 2009 meta-analysis of 23 studies concluded that ERT had small beneficial effect on muscle strength in postmenopausal women (41). In addition, a recent clinical trial in postmenopausal women demonstrated increased muscle mass and function parameters with ERT plus resistance training versus resistance training alone (42). However, data are lacking regarding women with POI (16). Our study’s finding of lower muscle mass including ALM and ALMI in women with s-POI or i-POI is consistent with results from Luo et al., which reported a lower muscle distributing coefficient of the lower limbs (MD) in 240 women with POI compared with peri- or postmenopausal women, not on ERT (43). This contrasts with the case-control study of 140 participants by Freitas et al., which did not observe any difference in the TFM or TLM in women with POI (44); however, the use of ERT for 18 months by these participants may have prevented muscle loss. Similarly, in the longitudinal analysis of our POI cohort, an increase in lean mass (ALM, ALMI) was observed in those with continued ERT use over the follow-up period. Multiple pathophysiological oestrogenic influenced mechanisms potentially contribute to reduced muscle mass including: reduced muscle turnover, defective contractility, increased free radical-mediated apoptosis and impaired bone-muscle cross signaling due to compromised skeletal health in POI (16). Overall, these findings highlight the deleterious effects of oestrogen deficiency on muscle health and potential prevention with ERT.

Limited data are available regarding fracture risk and prevalence in women with POI (7). A case-control study showed an increased prevalence of Colles’ fracture in women with early menopause (45) and findings of increased fracture risk with earlier menopause were also seen Women’s Health Initiative observational study (46). However, prevalent fractures in our study were similar in all three groups, likely explained by our small study size. As fracture risk is time-dependent, increased fracture prevalence in POI women may become significant later in life. We were unable to determine the type of fracture (fragility or traumatic); however, recent data in postmenopausal women indicates increased fracture risk with both fracture types (47).

The strengths of this study include: (i) the use of a novel tool, TBS, for fracture risk assessment, (ii) the presence of an age- and BMI-matched control group for cross-sectional analysis, and (iii) the availability of longitudinal skeletal and body composition data for POI participants. Despite providing important new insights into musculoskeletal health in women with POI, our study also has some limitations. These include: (i) retrospective data collection, (ii) inclusion of BMD and TBS data from one s-POI patient with an L1 fracture could have affected the BMD analysis. However, whether TBS is affected by mild to moderate compression fractures is still debated (48) (iii) small sample size of the longitudinal POI cohort that may have underestimated the effects of ERT on the longitudinal skeletal and body composition parameters; and (iv) lack of a longitudinal control group. The small sample sizes and the use of different ERT preparations by the same woman also precluded the analysis of bone and body composition parameters according to type of ERT and we therefore restricted our analysis to ‘interrupted’ or ‘continued’ ERT use. These considerations highlight the need for adequately powered prospective studies, as currently occurring in the United Kingdom, or randomised clinical trials to answer the important question as to the optimal ERT for bone and muscle health in women with POI. Given the limitation of a small cohort, we could not identify an association between TBS and fractures in women with POI.

## Conclusion

In this combined study of skeletal and body composition parameters, we demonstrate that lower BMD and spinal trabecular microarchitectural bone deficits, as assessed by TBS, are more common in POI women than healthy pre-menopausal women. Furthermore, these women are also at risk of losing muscle mass; ERT attenuates these changes. This study highlights the need to view bone and muscle health in unison, especially when bone microarchitectural and muscle assessments can be performed simultaneously with routine BMD assessment using commonly available DXA-based techniques.

## Data Availability Statement

The raw data supporting the conclusions of this article will be made available by the authors, without undue reservation.

## Ethics Statement

Ethical review and approval was not required for the study on human participants in accordance with the local legislation and institutional requirements. Written informed consent for participation was not required for this study in accordance with the national legislation and the institutional requirements.

## Author Contributions

NS, HN, FM, AV, and PE contributed to conception and design of the study. NS and HN organised the database. NS and HN performed the statistical analysis. NS prepared the manuscript. All authors contributed to manuscript revision, read, and approved the submitted version.

## Conflict of Interest

The authors declare that the research was conducted in the absence of any commercial or financial relationships that could be construed as a potential conflict of interest.

## Publisher’s Note

All claims expressed in this article are solely those of the authors and do not necessarily represent those of their affiliated organizations, or those of the publisher, the editors and the reviewers. Any product that may be evaluated in this article, or claim that may be made by its manufacturer, is not guaranteed or endorsed by the publisher.
